# Stability Analysis of Multi Process Parameters for Metal-Organic Chemical Vapor Deposition Reaction Cavity

**DOI:** 10.3390/molecules24050876

**Published:** 2019-03-01

**Authors:** Jian Li, Ziling Wu, Yifeng Xu, Yanli Pei, Gang Wang

**Affiliations:** 1 State Key Laboratory of Optoelectronic Materials and Technologies, Sun Yat-sen University, Guangzhou 51006, China; lijian66@mail.sysu.edu.cn (J.L.); wuzling@mail2.sysu.edu.cn (Z.W.); lnddxyf@126.com (Y.X.); peiyanli@mail.sysu.edu.cn (Y.P.); 2 Foshan Research Institute of Sun Yat-sen University, Foshan 528225, China

**Keywords:** MOCVD, film growth, process parameters, CFD, stable flow

## Abstract

The parameters for metal-organic chemical vapor deposition (MOCVD) processes significantly influence the properties of ZnO films, especially the flow stability of the chamber, which is caused by process parameters such as the shape of reaction chamber, the working pressure, the growth temperature, the susceptor rotational speed, the gas flow rate, and the nature of the carrier gas at inlet temperature. These parameters are the preconditions for the formation of high-quality film. Therefore, this study uses Ar as a carrier gas, diethylzinc (DEZn) as a Zn source, and H_2_O as an oxygen source and adopts the reaction mechanism calculated by quantum chemistry, which includes ten gas reactions and eight surface reactions. The process parameters of a specific reaction chamber model were analyzed based on the computational fluid dynamics method. This study also presents an accurate prediction of the flow regime in the reactor chamber under any operating conditions, without additional experiments, based on an analysis of a great quantity of simulation data. Such research is also significant for selecting the growth parameters relevant to production, providing a specific process growth window, narrowing the debugging scope, and providing a theoretical basis for the development of MOCVD equipment and process debugging.

## 1. Introduction

Zinc oxide is a wide-bandgap compound semiconductor material with excellent photoelectric properties [1,2,3]. As a new type of wide-bandgap semiconductor, ZnO has attracted increasing attention in recent years [4,5,6]. It has great value for development and application in ultraviolet (UV) laser diodes, UV light-emitting devices, thin-film transistors, and solar cells [7,8,9,10].

To make ZnO-based optoelectronic devices widely used, it is necessary to find high quality repeatable epitaxial film and hetero-structure growth technology. Because of the abundant sources of ZnO and the low growth temperature required, researchers have been able to grow and dope thin films by various methods for a long time. In terms of material quality, pulse laser deposition (PLD), molecular beam epitaxy (MBE), atomic layer deposition (ALD), and metal-organic chemical vapor deposition (MOCVD) have strong competitiveness [11,12,13,14,15,16]. However, this method is widely used in the fabrication of high-temperature superconductors (HTS) based on YBCO by MOCVD as well as conducting polymers (CPs), fabricated by a new technique derived from MOCVD called oxidative chemical vapor deposition (oCVD) [17,18]. Considering the factors of cost, output, and industrial application, MOCVD has many more remarkable characteristics. It can be used for large area growth, to precisely control the composition and thickness, has a high repeatability and growth rate, can cover complex substrate shapes, can rapidly switch gas paths to prepare steep multilayer interfaces, and is suitable for in-situ annealing [19,20,21,22].

The unique gas path design of a MOCVD reaction chamber makes the flow of gas complicated and invisible. Traditional design methods have become inadequate due to the cost of manpower, material resources, and time. Simulation has become a powerful tool with which to design a reaction chamber. GaN-MOCVD equipment has developed rapidly due to the wide application of GaN and other III-V materials and mature research mechanisms [23,24,25,26]. Researchers have a preliminary understanding of the gas flow and heat transfer process in the reaction chamber and have elucidated some basic relationships between the flow field, growth rate, deposition consistency, and process parameters in various MOCVD reaction chambers. The United States equipment company Veeco has carried out a lot of research on the design and process development of MOCVD reaction chambers. Kadinski et al. [27] considered that it was very important to use detailed three-dimensional geometric models for accurate predictions. The accuracy of the calculation model fully demonstrated the influence of process parameters and the effect of the geometric shape of the reaction chamber on growth rate and uniformity. The steady flow field was obtained by changing the MFC control parameters to suppress the eddy current in the reaction chamber during the growth of GaN/InGaN on the EMCORE D180 and E300 platforms; the stacking method was provided to adjust the uniformity. Mitrovic et al. [28,29] proposed a new “pressure-base speed” mapping method based on the typical flow in the vertical rotating disc MOCVD reaction chamber (VEECO E300 GaN), which could be used to stabilize the cavity flow in a wide range of process parameters. Subsequently, they combined the CFD simulation of the VEECO Turbo Disc Pioneer reaction chamber with the optimization of DOE test to determine the optimal geometric location for the gas inlet in the alkyl region [30]. This design provides a wide range of process conditions with which to obtain the most uniform deposition on the wafer. The predicted results agree well with the experimental results. This method greatly shortened the process development time and improved the uniformity of the growth and the efficiency of the alkyl efficiency. Liu et al. [31] used CFD technology to study the effect of the process parameters on the growth of zinc oxide films in a vertical MOCVD reaction chamber. Only the single-step total package chemical reaction mechanism was used. The results showed that the deposition rate was closely related to the operating parameters of the MOCVD reaction chamber. As the pressure in the chamber increased, the deposition rate increased and then decreased. With the increase in the inlet flow rate, the deposition rate first increased and then decreased. Chuang and Chen [32] studied a mathematical model and an optimal design for a horizontal MOCVD reaction chamber to grow a thin GaAs film. To improve the growth performance of thin films and meet high quality requirements, a data-driven optimization scheme based on a combination of neural networks and genetic algorithms was developed. The proposed scheme was applied to search for a set of operating conditions to optimize the growth rate and uniformity of thin films. The model agreed well with the experimental data, and the data-driven optimization scheme can be applied to optimize the process parameters of other horizontal MOCVD reaction chambers for long multicomponent semiconductors. Zhi Zhang et al. [33,34] used CFD to simulate a three-dimensional tight coupled (CCS) MOCVD reaction chamber considering fluid flow, heat transfer coupling, and chemical reactions in the reaction chamber. The growth rate and uniformity of the TMG:NH_3_ on the base surface was studied using the orthogonal test method. The TMG:NH_3_ content on the base surface was the main gas group that determined the growth rate and uniformity, and the growth rate increased as the total gas flow rate increased. An increase in nozzle height and inlet gas temperature showed that the film would be more uniform with a lower gas flow rate and higher inlet gas temperature.

There are few studies on the mechanisms of ZnO and 2–6 other II–VI oxide materials because they were developed recently, therefore, the development of oxide MOCVD equipment has been greatly limited [35,36,37,38,39,40,41]. In this study, the steady flow state of a three-dimensional ZnO-MOCVD reaction chamber using large-scale process parameters was studied using CFD based on the chemical reactions between DEZn and water-grown ZnO. The latter was developed using quantum chemistry and the MOCVD model was developed independently by MD-600B. In the process of ZnO growth using MOCVD, the pressure fluctuation range is typically controlled to within 1–20 torr. When Al-doped ZnO (AZO) grows, the growth chamber pressure can reach 60 torr or higher. The cavity speed is usually controlled between 500 RPM to 800 RPM and the substrate growth temperature ranges from 573 K to 773 K. This study reveals the relationship between inlet flow rate, inlet and substrate temperature, chamber pressure, base speed, and stable flow. It provides a specific process growth window for process operators using MO600B ZnO-MOCVD to grow ZnO materials, reduces the debugging range, and greatly reduces the loss of manpower and materials.

## 2. Problem Formulation

### 2.1. Governing Equations

The growth of ZnO film by MOCVD is a complex process that contains heat transfer, fluid flow, mass diffusion and chemical reactions. The following equations of continuity, momentum, energy and species transport in the work are expressed by Equations (1)–(5) [33,34,40].

(1) $$\nabla \cdot (\rho \vec{v})=0$$

(2) $$\nabla \cdot (\rho \vec{v}\cdot \vec{v})=\nabla \overset{=}{\tau }-\nabla p+\rho \vec{g}$$

where ρ is the density, $\vec{v}$ is velocity vector of the gas mixture, and $\overset{=}{\tau }$ is the shear stress tensor, respectively. p and $\vec{g}$ are the pressure and the gravitational acceleration.

(3) $$C_{p}\nabla \cdot \left(\rho \vec{v}T\right)=\nabla \cdot \left(k\nabla T\right)+\overset{N}{\underset{i=1}{\sum }}\frac{H_{i}}{M_{i}}\nabla \cdot \vec{J_{i}}-\overset{N}{\underset{i=1}{\sum }}\frac{H_{i,0}}{M_{i}}R_{i}$$

where $C_{p}$ is the specific heat capacity, k is the thermal conductivity, and T is the temperature. $H_{i},{\;H}_{i,0},{\;M}_{i}$ and $\vec{J_{i}}$ represent the molar enthalpy, enthalpy of formation, molar mass, and diffusion flux of species i, respectively.

(4) $$\nabla \cdot \left(\rho \vec{v}Y_{i}\right)=-\nabla \cdot \vec{J_{i}}+M_{i}\overset{K}{\underset{j=1}{\sum }}R_{j}^{i}$$

where $Y_{i}$ is the mass fraction of species i and $R_{j}^{i}$ is the net volumetric rate of creation of species i during reaction j. According to the Maxwell–Stefan theory, the diffusion flux vectors $\vec{J_{i}}$ can be given as follows:

(5) $$\vec{J_{i}}={\vec{j}}_{i}^{C}+{\vec{j}}_{i}^{T}=-\overset{N-1}{\underset{j=1}{\sum }}\rho D_{i,j}\nabla Y_{i}-D_{i}^{T}\frac{\nabla T}{T}$$

where concentration diffusion (${\vec{j}}_{i}^{C}$) is given by the concentration gradients and thermal diffusion (${\vec{j}}_{i}^{T}$) is given by the temperature gradients. $D_{i,j}$ is the mass diffusion coefficient for species i in the mixture and $D_{i}^{T}$ is the thermal diffusion coefficient.

### 2.2. Geometric Model Description

**Figure 1:**
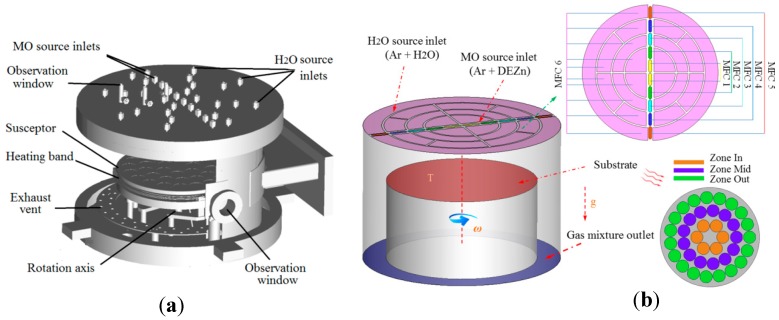
(**a**) 3D model diagram of the reaction chamber for the zinc oxide-metal-organic chemical vapor deposition (ZnO-MOCVD) prototype; (**b**) computational fluid dynamics simulation of the ZnO-MOCVD model and wafer distribution of the substrate surface.

A MD-600B ZnO-MOCVD reaction chamber was used for the simulations. Figure 1a shows the structure of the reaction chamber. To control the pre-occurrence of the reaction, an Ar flow carries DEZn in through an Zn source inlet; i is controlled by mass flow controllers 1–5 (MFC1–MFC5). Each MFC controls two sets of inlets, respectively, which is designed to be better applied to the adjustment of film uniformity. To be more specific, MFC1 controls the thickness of the zone in, MFC2 controls the thickness of the zone mid, MFC3 controls the thickness of the zone out, MFC4 and MFC5 play the role of MO air source supplement. Another Ar flow, controlled by MFC6, carries H_2_O through the surrounding O source inlet. The substrate base, which is rotating at high speed, is heated at a high temperature by the heating sheet. The specific calculation model is shown in Figure 1b. The Ar flows carrying DEZn and H_2_O are evenly mixed inside the cavity and then spread uniformly as the base rotates to form a ZnO film on the surface of the substrate.

### 2.3. Reaction Mechanisms

We used DEZn and oxygen (H_2_O) as sources for ZnO growth in a typical MOCVD process. The metal-organic precursor was DEZn and the oxygen source was H_2_O. The gases were injected from the showerhead to the chamber. Complex gas-phase and surface chemical reactions produced several intermediate products. We investigated the reaction mechanisms in detail in previous research [40]. Table 1 and Table 2 present the reaction mechanisms calculated using quantum chemistry [42], which include ten gas reactions and eight surface reactions.

The constant of the reaction rate is defined by the Arrhenius law [25,26],

(6) $$k_{k}=AT^{\beta }\exp\left(-\frac{E_{a}}{RT}\right),$$

where A, β, and $E_{a}$ are the pre-exponential factor, the temperature exponent, and the activation energy, respectively.

The growth rate of the film is determined by two factors [34,43]: The collision rate of the gas species on the surface and the reactants diffusing to the surface. During MOCVD growth, the mass transport factor is dominant, which means that the ZnO film deposition rate is controlled by the reactant transferring to the substrate surface. It is noted that the surface reaction rate is independent of the solid species. Hence, the mole rate of the kth surface reaction can be written as:

(7) $${\hat{R}}_{k}^{i}=F_{i}\times S$$

where $F_{i}$ is the mole flux of species i and S represents its sticking probability, ranging from zero to unity. The sticking probability lies in the range 0≤S≤1, where the two extremes correspond to no molecules being adsorbed or complete adsorption by all incident molecules, respectively [26].

## 3. Effect of Process Parameters on Flow Stability in Cavity

**Figure 2:**
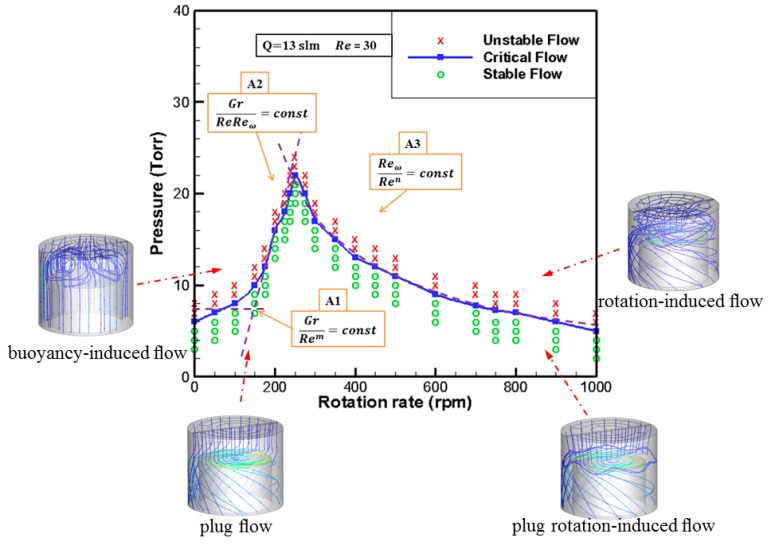
*P-ω* diagram for ZnO-MOCVD reaction chamber stability.

In the process of growing ZnO thin films by MOCVD, to obtain the required characteristic films, the process parameters are mainly dependent on the gas inlet flow rate, inlet gas temperature, substrate growth temperature, base rotation speed, and cavity pressure. As shown in Figure 2, there are four different flow states under different operating conditions [24]: Buoyancy-induced flow, plug flow, plug rotation-induced flow, and rotation-induced flow. The plug and plug rotation-induced flows are steady, while the buoyancy- and rotation-induced flows are unstable.

The characteristic dimensionless number array is used to describe the phenomena of gas crystal growth and transport as shown below.

Re is the characteristic number of the forced flow of the reacting fluid.

(8) $$Re=\rho_{0}\upsilon_{0}d/\mu_{0}$$

Gr is the characteristic number that reflects the degree of natural convection.

(9) $$Gr=\rho_{0}^{2}gH^{3}\left(t_{s}-t_{0}\right)/t_{0}\mu_{0}^{2}.$$

$Re_{\omega }$ is the characteristic number that reflects the forced convection caused by the rotational speed of the turntable.

(10) $$Re_{\omega }=\rho_{0}\omega r^{2}/\mu_{0}$$

where $\rho_{0}$ is the gas density, $\upsilon_{0}$ is the gas velocity, $\mu_{0}$ is the viscosity, d is the inlet diameter, g is the acceleration of gravity, H is the distance between the inlet and substrate, $t_{s}$ is the substrate temperature, $t_{0}$ is the temperature of the injected gas mixture, ω is the susceptor rotation rate, and r is the radius of the disk.

The relationship between pressure and rotational speed was studied under the conditions of fixed flow rate, inlet temperature and substrate temperature. When the speed was fixed, the pressure gradually increased. When the cavity becomes unstable, the pressure is the critical flow pressure. The critical pressure points were determined by a series of rotational speeds, and the steady flow curves were fitted. The flow below the curve is stable and the flow above the curve is unstable. The whole speed range can be approximately divided into three regions: In low-speed region A1 (ω < 100 RPM), the inlet flow leading to vortex suppression and the stability criterion for this area can be characterized by $Gr/Re^{m}$. In medium-speed region A2 (100 RPM < ω < 250 RPM), the stability criteria of this region may be characterized by $Gr/ReRe_{\omega }$. In high-speed region A3 (250 RPM < ω < 1000 RPM), this area can be used as the stability criterion, characterized by $Re_{\omega }/Re^{n}$. The results indicate that the peak value of critical pressure is around 250 RPM, which is the critical speed to distinguish buoyancy-driven flow from rotation-driven flow. This is the result of the interaction of multi-process parameters.

Based on the process parameters with different flow rates, different inlet temperatures, and different susceptor temperatures, the flow prediction map of the reaction chamber is proposed without additional numerical simulation or experimentation. Such a study is also of great significance to the selection of growth parameters in production. It provides a specific growth process window to maintain laminar flow stability, reduces the debugging scope and saves time and effort.

### 3.1. Effect of Inlet Flow on Stability of Reaction Chamber

**Figure 3:**
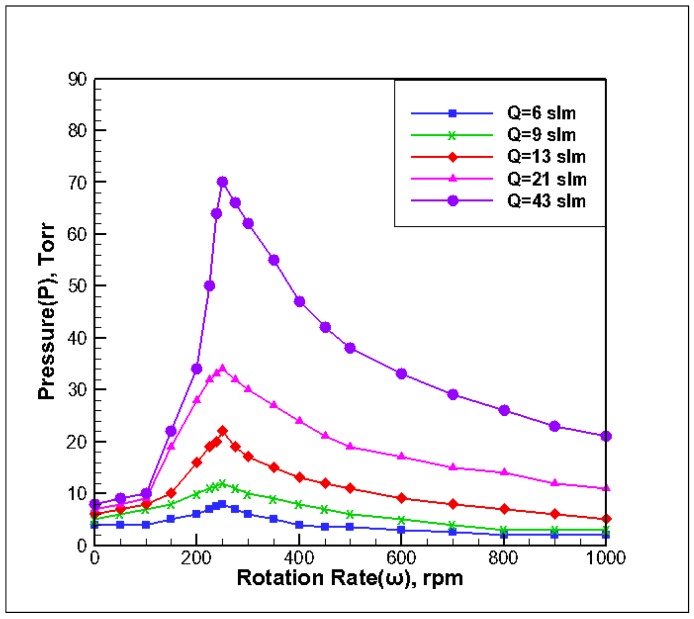
*P-ω* stability map under different inlet flow rates.

The substrate temperature of the reaction chamber (T_susceptor_) was 673 K, and the inlet gas temperature was T_in_ = 300 K. According to the principle of stability determination, the pressure and speed were adjusted to determine the *P-ω* stability map. The simulation results under different inlet flow rates were processed according to the stability principle, based on the stability judgment method shown in Figure 2, and fitted into five stability extremum curves, as shown in Figure 3. The following observations can be made from the figure:

(1) With the increase in the gas inlet flow rate, the critical operating pressure in the corresponding curved areas changed significantly. The curve with the large flow rate is above the curve with the small flow rate. Because increasing the Re value increases the reaction gas flow, forced convection increases from top to bottom, and the buoyancy and rotational flow are restrained. That is to say, a wider pressure growth window was needed to maintain stability under a larger gas inlet flow rate, and the range of operating pressure also increased when the rotational speed of the base was the same.

(2) With the increase in the gas inlet flow rate, the corresponding critical operating pressures A1, A2, and A3 have corresponding changes in the speed range. The rotational speed of the A1 region gradually decreased with the increase of the gas inlet flow rate, and the change in the critical pressure value was relatively small. The rotational speed of the A2 region increased with the increase in the gas inlet flow rate. For a rotational speed ranging from 100 rpm to 300 rpm, the maximum allowable pressure can be obtained by translating the peak value of the operating pressure to the right, and the slope of the curve in this region is larger. It can be seen that the critical pressure of the stable point varied greatly when the inlet flow rate was increased. The rotational speed in the C region decreased with an increase in the gas inlet flow and the rate of change of critical pressure decreased. The increase in gas flow mainly affected regions A2 and A3, and the influence on region A1 was relatively weak.

### 3.2. Effect of Inlet Temperature on Stability of Reaction Chamber

**Figure 4:**
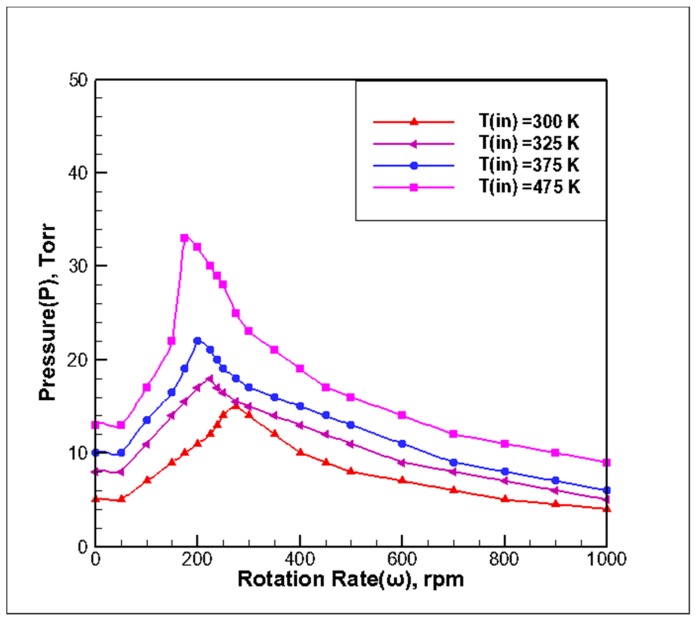
*P-ω* stability map at different inlet temperatures.

The temperature of the reaction chamber base (T_susceptor_) was 673 K and the gas inlet flow rate was Q = 13 slm. The *P-ω* stability map of T_in_ with different inlet temperatures was determined by adjusting the pressure and rotational speed according to the principle of stability determination. According to the stability principle, the simulation results of different inlet temperatures can be fit to four extreme value curves of stable points, as shown in Figure 4, according to the processing method shown in Figure 2. The following observations can be made from the curve:

(1) With the increase in the inlet gas temperature, the maximum pressure required for maintaining the stability of the flow field in the reaction chamber increases. The velocity increases with the increase in the inlet gas temperature, and forced convection from top to bottom increases, which restrains the buoyancy and rotational flow. The curve of high inlet temperature is above the curve of low inlet temperature. When the inlet temperature was increased, Gr was reduced, so the inlet velocity required to suppress natural convection was also reduced. Because υ∝1/P, the critical value of stability pressure will be increased with the increase of inlet temperature. A wider pressure growth window was required to maintain stability in the case of a higher inlet temperature, and the range of operating pressure also increased when the rotational speed of the base was the same.

(2) With the increase in the inlet gas temperature, the corresponding critical operating pressures in the A1, A2, and A3 regions experienced some changes in their speed range. The rotational speed in the A1 region gradually decreased with the increase in the inlet gas temperature, and the change in critical pressure is obvious. The rotational speed in the A2 region gradually decreased with the increase in the inlet gas temperature. For a rotational speed varying from 200 rpm to 300 rpm, the peak pressure during the operation shifts to the left to obtain the maximum allowable pressure. The slope of the curve increased, that is, the inlet gas temperature increased and the rate of change in the critical pressure was greater. The rotational speed in the A3 region increased with the increase in the inlet temperature. It can be seen that an increase in the inlet gas temperature mainly affected the A1 and A2 regions, while the effect on the A3 region was relatively weak.

### 3.3. Effect of Substrate Growth Temperature on the Stability of the Reaction Chamber

**Figure 5:**
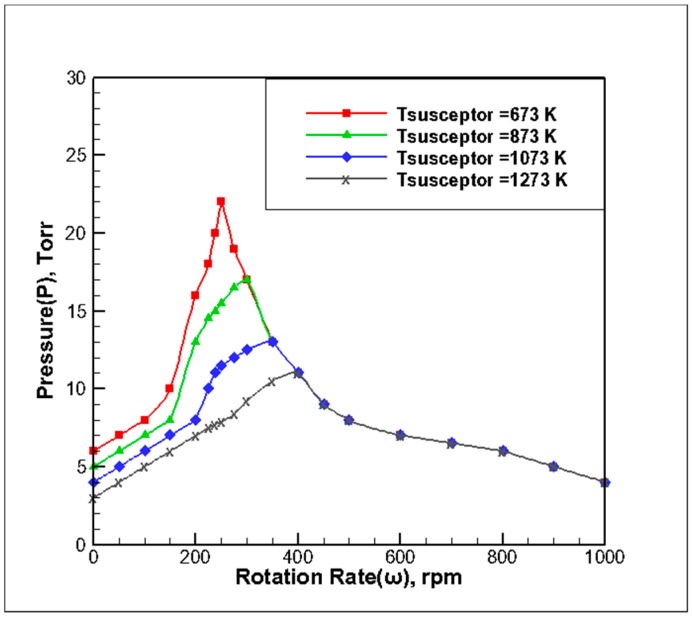
Stability map of *P-ω* at different substrate growth temperatures.

By setting the reaction chamber inlet temperature (T_in_) to 300 K and the gas inlet flow (Q) to 13 slm at different base temperatures, respectively, the *P-ω* diagram was determined by adjusting the pressure and rotational speed according to the principle of stability determination. The simulation results at different substrate temperatures are based on the stability principle, which is based on the processing method shown in Figure 2. The four extreme points of stability, as shown in Figure 5, were synthesized. The following observations can be made from the curve:

(1) With the increase in the substrate growth temperature, the maximum pressure required to maintain field stability was smaller. As the substrate gas temperature increased, the thermal buoyancy increased with the increase in Gr, resulting in the more obvious unstable flow state. In regions A1 and A2, the operating pressure curve at a high substrate temperature was located below the substrate temperature curve. In the A3 region, the curve of the substrate temperature was basically coincidental with the curve of low substrate temperature. When the susceptor temperature rose, Gr increased, and a larger inlet velocity was needed to suppress natural convection. The critical value of stable pressure decreased with the increase of susceptor temperature because υ∝1/P.

(2) With the increase in substrate temperature, there were some changes in the speed range of the critical operating pressure in the A1, A2, and A3 regions. The rotational speed in the A1 region decreased with the increase in substrate temperature, and the change in the critical pressure value is more obvious. The rotational speed in the A2 region increased with the increase in substrate temperature. From 200 rpm to 400 rmp, the maximum allowable pressure can be obtained by translating the peak value of the operating pressure to the right, and the slope of the curve in this area increases. It can be seen that when the substrate growth temperature decreased, the rate of change in the critical pressure of the stable point was larger. The rotational speed in the A3 region decreased with an increase in substrate temperature, and the rate of change in the critical pressure for increasing rotational speed remained basically unchanged. It can be seen that increasing the substrate temperature mainly affected the A1 and A2 regions but had little effect on high-speed regions, such as the A3 region.

## 4. Conclusions

Using MD600B ZnO-MOCVD as the prototype machine and the transport-reaction model of ZnO growth by DEZn and H_2_O, the large-scale *P-ω* stability maps were established through high-speed rotating disc reactions. The growth window of ZnO-MOCVD can be enlarged by increasing the inlet gas flow rate and inlet gas temperature and decreasing the substrate growth temperature. With a decrease in the inlet flow rate and an increase in the substrate temperature and inlet gas temperature, the speed regulation range in the A2 region decreases. With a decrease in the inlet flow rate and an increase in the substrate temperature and gas inlet temperature, the speed regulation range in the A3 region increases. The A1 region is less affected by the change in process parameters. This theory has played a guiding role in the growth and debugging of MB-600D ZnO-MOCVD by technicians and the development of good ZnO epitaxial films.

## Figures and Tables

**Table 1:** Gas-phase reaction pathways and kinetic parameters of diethylzinc (DEZn) hydrolysis.

No.	Reaction	A	$E_{a}$
G1	Zn(CH_2_CH_3_)_2_ + H_2_O → Zn(CH_2_CH_3_)_2_·H_2_O	Coll.	0
G2	Zn(CH_2_CH_3_)_2_·H_2_O → C_2_H_6_ + Zn(CH_2_CH_3_)OH	2.96 × 10^12^	28.2
G3	Zn(CH_2_CH_3_)OH + H_2_O → Zn(OH)_2_ + C_2_H_6_	1.1 × 10^14^	39.8
G4	Zn(CH_2_CH_3_)_2_ + 2H_2_O → H_2_O ·Zn(CH_2_CH_3_)_2_·H_2_O	Coll.	0
G5	H_2_O ·Zn(CH_2_CH_3_)_2_·H_2_O →C_2_H_6_·Zn(CH_2_CH_3_)OH·H_2_O	8.97 × 10^12^	28.3
G6	C_2_H_6_·Zn(CH_2_CH_3_)OH·H_2_O → Zn(OH)_2_ + 2C_2_H_6_	8.03 × 10^13^	38.6
G7	3Zn(OH)_2_ → Zn_3_(OH)_6_	Coll.	0
G8	Zn_3_(OH)_6_ → Zn_3_O_5_H_4_ + H_2_O	2.42 × 10^13^	39.2
G9	Zn_3_O_5_H_4_ → Zn_3_O_4_H_2_ + H_2_O	1.67 × 10^12^	30.6
G10	Zn_3_O_4_H_2_ → Zn_3_O_3_ + H_2_O	1.08 × 10^12^	17.3

E_a_: Activation energy (kcal/mol), A: Pre-exponential factor (1/s).

**Table 2:** Surface reaction pathways and kinetic parameters of for diethylzinc (DEZn) hydrolysis.

No.	Reaction	A	$E_{a}$
S1	Zn(CH_2_CH_3_)_2_·H_2_O→ZnO+2C_2_H_6_	s = 1	8.36
S2	H_2_O·Zn(CH_2_CH_3_)_2_·H_2_O→ZnO+2C_2_H_6_+H_2_O	s = 1	8.36
S3	Zn(CH_2_CH_3_)OH → ZnO + C_2_H_6_	s = 1	0
S4	C_2_H_6_·Zn(CH_2_CH_3_)OH·H_2_O→ ZnO + 2C_2_H6 + H_2_O	s = 1	0
S5	Zn (OH)_2_ → ZnO + H_2_O	s = 1	0
S6	Zn_3_(OH)_6_ → 3ZnO + 3H_2_O	s = 1	0
S7	Zn_3_O_5_H_4_ → 3ZnO + 2H_2_O	s = 1	0
S8	Zn_3_O_4_H_2_ → 3ZnO + H_2_O	s = 1	0

S = a free surface site. s = a sticking probability of unity (1).
